# Balanced versus chloride-rich solutions for fluid resuscitation in brain-injured patients: a randomised double-blind pilot study

**DOI:** 10.1186/cc12686

**Published:** 2013-04-19

**Authors:** Antoine Roquilly, Olivier Loutrel, Raphael Cinotti, Elise Rosenczweig, Laurent Flet, Pierre Joachim Mahe, Romain Dumont, Anne Marie Chupin, Catherine Peneau, Corinne Lejus, Yvonnick Blanloeil, Christelle Volteau, Karim Asehnoune

**Affiliations:** 1 Pôle Anesthésie-Réanimations, Service d'anesthésie réanimation Hôtel-Dieu, CHU Nantes, F-44000 Nantes, France; 2 Pôle Anesthésie-Réanimations, Service d'anesthésie réanimation Hôpital Laennec, CHU Nantes, F-44000 Nantes, France; 3 Pôle Anesthésie-Réanimations, Service de réanimation médicale polyvalente, CHU Nantes, F-44000 Nantes, France; 4 Pôle Pharmacie, Service HOTEL-DIEU-Essais cliniques, CHU Nantes, F-44000 Nantes, France; 5 Cellule de Biostatistiques-Cellule de promotion à la recherche clinique, CHU Nantes, F-44000 Nantes, France

## Abstract

**Introduction:**

We sought to investigate whether the use of balanced solutions reduces the incidence of hyperchloraemic acidosis without increasing the risk for intracranial hypertension in patients with severe brain injury.

**Methods:**

We conducted a single-centre, two-arm, randomised, double-blind, pilot controlled trial in Nantes, France. Patients with severe traumatic brain injury (Glasgow Coma Scale score ≤8) or subarachnoid haemorrhage (World Federation of Neurosurgical Society grade III or higher) who were mechanically ventilated were randomised within the first 12 hours after brain injury to receive either isotonic balanced solutions (crystalloid and hydroxyethyl starch; balanced group) or isotonic sodium chloride solutions (crystalloid and hydroxyethyl starch; saline group) for 48 hours. The primary endpoint was the occurrence of hyperchloraemic metabolic acidosis within 48 hours.

**Results:**

Forty-two patients were included, of whom one patient in each group was excluded (one consent withdrawn and one use of forbidden therapy). Nineteen patients (95%) in the saline group and thirteen (65%) in the balanced group presented with hyperchloraemic acidosis within the first 48 hours (hazard ratio = 0.28, 95% confidence interval [CI] = 0.11 to 0.70; *P *= 0.006). In the saline group, pH (*P *= .004) and strong ion deficit (*P *= 0.047) were lower and chloraemia was higher (*P *= 0.002) than in the balanced group. Intracranial pressure was not different between the study groups (mean difference 4 mmHg [-1;8]; *P *= 0.088). Seven patients (35%) in the saline group and eight (40%) in the balanced group developed intracranial hypertension (*P *= 0.744). Three patients (14%) in the saline group and five (25%) in the balanced group died (*P *= 0.387).

**Conclusions:**

This study provides evidence that balanced solutions reduce the incidence of hyperchloraemic acidosis in brain-injured patients compared to saline solutions. Even if the study was not powered sufficiently for this endpoint, intracranial pressure did not appear different between groups.

**Trial registration:**

EudraCT 2008-004153-15 and NCT00847977

The work in this trial was performed at Nantes University Hospital in Nantes, France.

## Introduction

Brain injuries remain a major concern for public health services, particularly because of the high mortality rate and long-term disabilities that result [1]. In the early stages of caring for brain-injured patients, therapies are focused on minimising secondary brain injuries that are centrally involved in determining outcomes [2]. Intracranial hypertension (ICH) is the most frequent cause of death and secondary brain insults after brain injury [3]. The maintenance of adequate cerebral perfusion pressure (CPP), which is associated with control of intracranial pressure (ICP), is the cornerstone of treating the ion deficit associated with brain ischaemia in brain-injured patients. Infusion of hypo-osmotic solutions, which increases cerebral swelling, should be avoided after brain injury [4,5]. Current recommendations are to use isotonic solutions in patients with severe brain injury [6,7], with isotonic sodium chloride (0.9% saline solution) being the mainstay of therapy. Isotonic sodium chloride solutions induce hyperchloraemic metabolic acidosis and have side effects including haemostatic alterations, cognitive dysfunction and ileus [8].

Hyperchloraemia is relatively common in critically ill patients, and it is now commonly accepted that chloride-rich fluids are the primary cause of hyperchloraemic acidosis in critically ill patients [9]. In a before-after study, a chloride-restrictive strategy was associated with a significant decrease in renal failure in critically patients and significantly affected electrolyte and acid-base status [10]. In a *post hoc *analysis of a retrospective study in TBI patients receiving isotonic sodium chloride solutions for basal infusion [11], 65% of the patients experienced hyperchloraemia. Chloride channels regulate cell oedema [12], and it could be hypothesised that dyschloraemia contributes to brain swelling.

Isotonic balanced solutions are now available and include crystalloids as well as hydroxyethyl starch (HES) solutions. In these isotonic solutions, the use of malate and acetate allows the reduction of chloride concentration while ensuring isotonicity. Balanced solutions could thus reduce the incidence of hyperchloraemic metabolic acidosis. Balanced solutions decrease the rate of hyperchloraemic acidosis in healthy volunteers [13,14] and during perioperative care compared with saline solutions [15-17]. To date, no data regarding isotonic balanced solutions for brain-injured patients have been published, and use of these solutions is therefore not recommended in this setting. The use of a balanced solution would appear to be attractive in brain-injured patients who are prone to ion homeostasis disruption, notably through hormonal dysfunction such as diabetes insipidus or cerebral salt-wasting syndrome or through alterations of chloride-dependent channels such as the NKCC1 transporter [18,19]. We postulated that infusion of isotonic balanced solutions instead of saline solutions would diminish the incidence of hyperchloraemic acidosis without increasing ICP in patients with severe brain injury hospitalised in the ICU.

## Materials and methods

### Ethical approval and study design

This randomised, double-blind, parallel, controlled study was approved by the Institutional Review Board of Tours, France (Région Centre, Ouest-1) (Trial registration: EudraCT 2008-004153-15 and NCT00847977). Patients were enrolled after their next-of-kin provided written informed consent. Retrospective consent, when available, was obtained from patients. Patients were enrolled from October 2008 to October 2010, when recruitment was completed in three ICUs of the Nantes University Hospital.

### Patient population

Patients with severe traumatic brain injury (TBI) (Glasgow Coma Scale score ≤8) on mechanical ventilation within the first 12 hours after brain injury were included. During recruitment, we refined the eligibility criteria by including patients with subarachnoid haemorrhage (SAH) at World Federation of Neurosurgical Societies (WFNS) grade III or worse (amendment of 26 July 2010). Exclusion criteria were multiple trauma, pregnancy, azotaemia above 200 μmol/L, kalemia less than 2.5 mmol/L, calcaemia less than 1.8 mmol/L, HES hypersensitivity, haemophilia or von Willebrand disease. Patients were also excluded when hypertonic saline solutions (HSSs) were used prior to inclusion or within the first 6 hours of the study start.

### Randomisation

Patients were randomised in a 1:1 ratio to either the balanced group (allocated solutions, crystalloids: Isofundine/HES: Tetraspan; B Braun Medical, Melsungen, Germany) or the saline group (allocated solutions, crystalloids: 0.9% saline solution/HES: HEAfusine, B Braun Medical) (Table 1). Randomisation was performed in blocks of eight by a computerised number generator list provided by a statistician not involved in the determination of eligibility or in the assessment of outcomes. The study packs were sealed in identical sequentially numbered boxes containing the entire treatment for each patient. Each "Iso-TC treatment packet" contained Isofundine or 0.9% saline solution (sheath labelled "crystalloid"), Tetraspan or HEAfusine (sheath labelled "HES"), and a sheet was also provided for the administration schedule. Patients, investigators, members of the monitoring board and medical and nursing staff were unaware of the patients' treatment assignment.

**Table 1:** Electrolyte composition of studied fluids.

	Saline group	Balanced group
**Crystalloid solutions**	**0.9% saline solution**	**Isofundine**

Sodium (mmol/L)	153	140
Potassium (mmol/L)	0	4.0
Calcium (mmol/L)	0	2.5
Magnesium (mmol/L)	0	1.0
Chloride mmol/L)	153	127
Acetate (mmol/L)	0	24
Malate (mmol/L)	0	5.0
pH	4 to 7	4.6 to 5.4
Theoretical osmolarity (mOsmol/L)	306	304
Acid titre	<2	<2

**Hydroxyethyl starch solutions**	**HEAfusine**	**Tetraspan**

Poly(O-2-hydroxyethyl) starch (g/L)	60	60
Molar substitution	0.5	0.42
Average molecular weight (Da)	200,000	130,000
Sodium (mmol/L)	153	140
Potassium (mmol/L)	0	4.0
Calcium (mmol/L)	0	2.5
Magnesium (mmol/L)	0	1.0
Chloride (mmol/L)	153	118
Acetate (mmol/L)	0	24
Malate (mmol/L)	0	5.0
pH	4 to 7	5.6 to 6.4
Theoretical osmolarity (mOsmol/L)	310	296
Acid titre	<2	<2

### Conduct of the study

Administration of the studied solutions began immediately after patient admission and lasted 48 hours. The attributed crystalloid was administered as a continuous intravenous infusion (30 ml/kg/day). The attending physician could administer optional boli (20 ml/kg of the attributed crystalloid or 10 ml/kg of the attributed HES over 20 minutes). Apart from blood products, other intravenous fluids were not allowed during the first 48 hours. After the 48th hour, fluid infusions were not controlled.

### General care for brain-injured patients

Brain-injured patients were mechanically ventilated and were sedated with fentanyl and midazolam (0.9% saline solution as drug-carrier solution). Patients were kept in a semirecumbent position. Continuous enteral nutrition was initiated 24 hours after brain injury [20]. The rate of enteral nutrition (Fresubin; Fresenius-Kabi, France) was increased every 8 hours until it reached 83 ml/h (2,000 kcal/day) (see Additional file 1 for full description). Parenteral nutrition was started on day 7 in patients intolerant to gastric feeding. Secondary brain injuries were prevented by avoiding hypoxaemia and anaemia (haemoglobin <10 g/dl), maintaining body temperature between 36.0°C and 37.0°C, ensuring normoglycaemia and normocapnia (between 4.6 and 5.5 kPa). ICP was monitored with an intraparenchymal probe placed in the most affected side (Codman; Johnson & Johnson, Raynham, MA, USA) in patients with severe brain injuries who had abnormal computed tomography (CT) scans and were considered at increased risk of ICH [21]. Extraventricular drainage was used in case of hydrocephalus detected on CT scans. Patients were monitored by invasive arterial pressure and mean arterial pressure (MAP) was measured up to the brain for the calculation of CPP. CPP was maintained above 60 mmHg with boli of the attributed isotonic solutions (crystalloid or HES; see Table 1) and continuous infusion of norepinephrine (diluted in 0.9% saline solution). Mannitol (bolus of 0.5 g/kg repeatable once in case of poor ICP control, ICP >20 mmHg, after 30 minutes; maximum dose: 1 g/kg) was used to control episodes of ICH. When control of ICH was poor, sodium thiopental was used with a loading dose (2 to 3 mg/kg) followed by continuous administration (2 to 3 mg/kg/h) adapted to ICP evolution and to serum level monitoring (blood level of thiopental between 20 and 30 μg/ml). A continuous infusion of HSS (20% saline solution) was started in case of refractory ICH [11]. When control of ICH was poor, decompressive craniectomy or therapeutic hypothermia was discussed with the neurosurgical team. The evolution of brain injuries was assessed by CT within the first 72 hours after brain injury.

### Data handling

The following data were recorded: general characteristics, including demography, initial GCS score, WFNS grade, time from tracheal intubation to study inclusion, vasopressor therapy, fluid challenges and surgical procedures prior to inclusion. Natraemia, chloraemia, kalaemia, magnesemia, phosphatemia, ionized calcaemia, azotaemia, albuminaemia, osmolarity, lactataemia, arterial gases and haematocrit were measured immediately before and at 6, 12, 24, 36 and 48 hours after starting the treatment. The total volume of fluid administered and the evolution of ICP were recorded during the study period (48 hours). Episodes of ICH, modifications on the control CT (bleeding, herniation or brain swelling), osmotherapy and/or barbiturate use, transfusion, vasopressor use, time to achieve more than 50% of goal calories of enteral nutrition, duration of mechanical ventilation, length of ICU stay and mortality rate were also recorded in the ICU. Safety was assessed by recording adverse events.

### Definitions

Strong ion difference (SID) was defined as (Na^+ ^+ K^+ ^+ Ca^2+ ^+ Mg^2+^) - (Cl^- ^+ lactate) mEq/L [22]. Hyperchloraemic metabolic acidosis was defined as SID below 40 mEq/L associated with chloraemia above 108 mmol/L according to local laboratory normal ranges.

### Endpoints

The primary endpoint was the occurrence of hyperchloraemic metabolic acidosis within 48 hours. The secondary outcomes were electrolyte status, ICP, rate of ICH episodes, volume of intravenous fluid, duration of vasopressor therapy, duration of mechanical ventilation, length of ICU stay and death in the ICU.

### Statistical analysis

To the best of our knowledge, the incidence of hyperchloraemic acidosis in brain-injured patients has not been documented to date. We have thus performed a *post hoc *analysis of the chloraemia values collected in a study of TBI patients with ICH receiving HSS [11]. We found a 65% incidence of hyperchloraemia within the first four days in the ICU before any HSS infusion. The sample size needed to detect a 45% decrease in the incidence of hyperchloraemic acidosis, assuming a basal rate of 65% in a two-sided test performed with a statistical power of 85% and an α risk of 0.05, was 20 patients in each group in this pilot study. Taking into account exclusions, and in an attempt to keep the power of the study, 42 patients (21 patients in each group) were included.

The full analysis set (FAS) of patients was the primary population used for statistical analysis of efficacy (per-protocol analysis) and was defined as all randomised patients treated with the study drug who did not receive forbidden therapy (HSS infusion). All randomised patients (the intention-to-treat (ITT) population) were analysed for the primary outcome and safety variables.

We first verified that in all patients the incidence of hyperchloraemic acidosis at 48 hours was significantly decreased in the balanced group compared with the control group using Fisher's exact test. Six patients experienced hyperchloraemic acidosis prior to inclusion (four in the saline group and two in the balanced group). We therefore decided *a posteriori *to perform two complementary sensitivity analyses. The first excluded patients with preexisting hyperchloraemic acidosis, the second censored the preinclusion biological values (SID, chloraemia) and the third consisted of evaluating the effect of balanced solutions on the primary outcome on the basis of a logrank test.

For secondary outcomes, linear mixed models were used with group effect, time effect and interaction between time effect and group effect. We first investigated the interaction between time effect and group effect. For the values with no significant interaction, the mean difference between groups within the study period was provided. For the value with a significant interaction between time effect and group effect, comparisons were performed independently and P values were calculated at each time point. Residual analysis was used to assess the appropriateness of the models (including normality and homoscedasticity). Nonparametric data are expressed as medians and interquartile ranges (IQRs). Categorical data are expressed as numbers and percentages. χ2 test, Fisher's exact test and Wilcoxon rank-sum test were used as appropriate.

A subgroup analysis considering severe TBI patients was performed a posteriori using the same analytical strategy. Regarding ICP evolution, subgroup analysis considering the 15 patients with ICH was performed. All statistical tests were two-sided. Statistical analyses were performed using SAS 9.1 statistical software (SAS Institute, Cary, NC, USA).

## Results

### Study population

**Figure 1:**
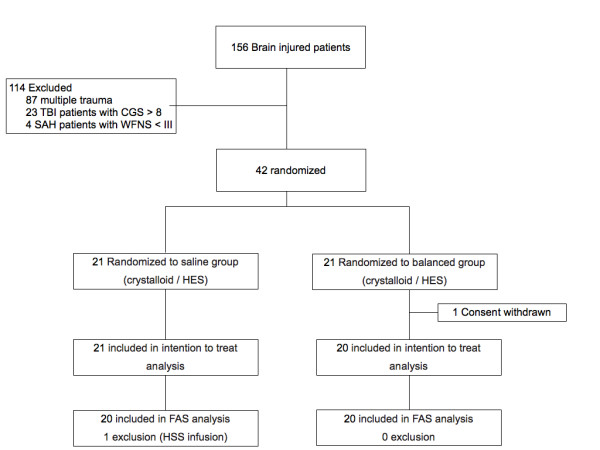
**Flowchart of the study**. GCS: Glasgow Coma Scale score, FAS: full analysis set; HES: hydroxyethyl starch; HSS: hypertonic saline solution, SAH: subarachnoid haemorrhage, TBI: traumatic brain injury, WFNS: World Federation of Neurological Societies.

Of the 42 patients included, 41 were included in the ITT analysis (one consent withdrawal) and 40 were included in the FAS analysis (exclusion of one patient who received HSS infusion within the first six hours; Figure 1). Demographic data are provided in Table 2 (see Additional file, Table S1, for the demographics of the FAS population). The total volume of fluid infusion was not altered by study group (Table 3). The total amount of chloride infusion was lower in the balanced group than in the saline group (median 744 mmol (IQR = 572 to 952) and median 918 mmol (IQR = 689 to 1,148), respectively; *P *= 0.014) (Table 3). Two patients in the saline group and one patient in the balanced group received one bolus of 500 ml of colloid diluted in a saline solution (Gelofusine; B Braun Medical) out of the study protocol, and data from these patients were kept in the statistical analysis.

**Table 2:** Baseline characteristics^a^

Characteristics	Intention-to-treat population		TBI patients	
	
	Saline (*N *= 21)	Balanced (*N *= 20)	Saline (*N *= 18)	Balanced (*N *= 18)
Age (years), median (IQR)	51 (25 - 68)	49 (26 - 75)	47 (28-57)	49 (27-77)
Male, *N *(%)	15 (71)	17 (85)	13 (72)	16 (89)
Diagnosis, *N *(%)				
Traumatic brain injury	19 (90)	18 (90)	/	/
Subarachnoid haemorrhage	2 (10)	2 (10)		
Glasgow Coma Scale score on scene, median (IQR)	8 (7 - 9)	7 (6 - 9)	7 (6-8)	7 (6-8)
Surgical procedures, *N *(%)				
Haematoma/contusion evacuation	9 (43)	4 (20)	8 (44)	4 (22)
Extraventricular drainage	2 (10)	2 (10)	0	0
Transfusion, median (IQR)				
Red blood cells, units	4 (19)	3 (15)	4 (22)	3 (17)
Fresh frozen plasma, units	3 (14)	0	3 (17)	0
Norepinephrine infusion on admission, *N *(%)	8 (38)	7 (35)	7 (39)	7 (39)
Fluid infusion prior to inclusion, median (IQR)				
Crystalloids (NaCl 0.9%), ml	1000 (500 -1000)	1000 (500 - 1500)	500 (500 -1000)	1000 (500 -1500)
Colloids, ml	0 (0 - 500)	0 (0 - 500)	0 (0 - 500)	0 (0 - 500)
Time from brain injury to inclusion (hours) median (IQR)	5 (3 - 7)	5 (4 - 12)	4 (3-6)	5 (4-6)
Biological status upon inclusion				
Hyperchloraemic acidosis, *N *(%)	4 (19)	2 (10)	4 (22)	2 (11)
Osmolarity (mOsm/L), median (IQR)	303 (295 - 319)	302 (296 - 319)	306 (299-328)	306 (300-319)
Natraemia (mmol/L), median (IQR)	140 (138-142)	139 (137-141)	140 (138-141)	139 (137-141)
Chloraemia (mmol/L), median (IQR)	106 (104 - 110)	106 (101 - 107)	106 (104-111)	106 (100-107)
Kalemia (mmol/L), median (IQR)	3.6 (3.4 - 3.9)	3.7 (3.5 - 4.1)	3.7 (3.4-3.9)	3.7 (3.5-4.1)
Ionized calcaemia (mmol/L), median (IQR)	1.10 (1.07 - 1.14)	1.11 (1.07 - 1.18)	1.10 (1.08-1.12)	1.11 (1.06-1.18)
Magnesemia (mmol/L), median (IQR)	0.86 (0.72 - 0.95)	0.81 (0.75 - 0.88)	0.82 (0.76-0.92)	0.83 (0.73-0.92)
Phosphoremia (mmol/L), median (IQR)	0.86 (0.67 - 1.07)	0.85 (0.74 - 1.14)	0.88 (0.70-1.10)	0.82 (0.73-1.13)
Lactataemia (mmol/L), median (IQR)	1.4 (1.1 - 2.1)	1.7 (1.1 - 2.7)	1.5 (1.2-2.2)	2.0 (1.1-2.7)
Azotaemia (μmol/L), median (IQR)	66 (56-73)	67 (58-71)	65 (56-72)	67 (60-71)
Albuminaemia (g/L), median (IQR)	36 (32 - 41)	37 (34 - 39)	34 (32-41)	36 (34-39)
pH, median (IQR)	7.38 (7.33 - 7.44)	7.39 (7.32 - 7.45)	7.36 (7.31-7.41)	7.39 (7.31-7.45)
SID (mmol/L), median (IQR)	39 (35 - 42)	39 (39 - 41)	40 (37-41)	39 (37-41)

^a^IQR: interquartile range; TBI: traumatic brain injury; Strong Ion Difference (SID) = (Na + K + Ca + Mg) - (Cl + lactate).

**Table 3:** Fluid administration within the first 48 hours

		Median (IQR)		
		**Saline group** **(*N *= 20)**	**Balanced group** **(*N *= 20)**	***P*-value**

Na administration (mmol)	H0 **to **H48	918 (689 to 1,148)	840 (630 to 1,050)	0.228
Cl administration (mmol)	H0 to H48	918 (689 to 1,148)	744 (572 to 952)	0.014

Crystalloids (ml)	H0 to H6	2,000 (1,000 to 2,000)	1,000 (500 to 2,000)	0.255
	H6 to H24	2,000 (1,500 to 2,000)	1,500 (1,500 to 2,000)	0.530
	H24 to H48	2,000 (1,500 to 2,000)	2,000 (1,500 to 2,000)	0.755
	H0 to H48	5,000 (4,500 to 6,000)	5,000 (4,000 to 6,000)	0.448

Hydroxyethyl starch solutions (ml)	H0 to H6	0 (0 to 500)	0 (0 to 500)	0.613
	H6 to H24	0 (0 to 500)	0 (0 to 500)	0.563
	H24 to H48	0 (0 to 0)	500 (0 to 1,000)	0.060
	H0 to H48	500 (0 to 1,500)	1,000 (500 to 1,500)	0.228

### Efficacy outcomes

**Figure 2:**
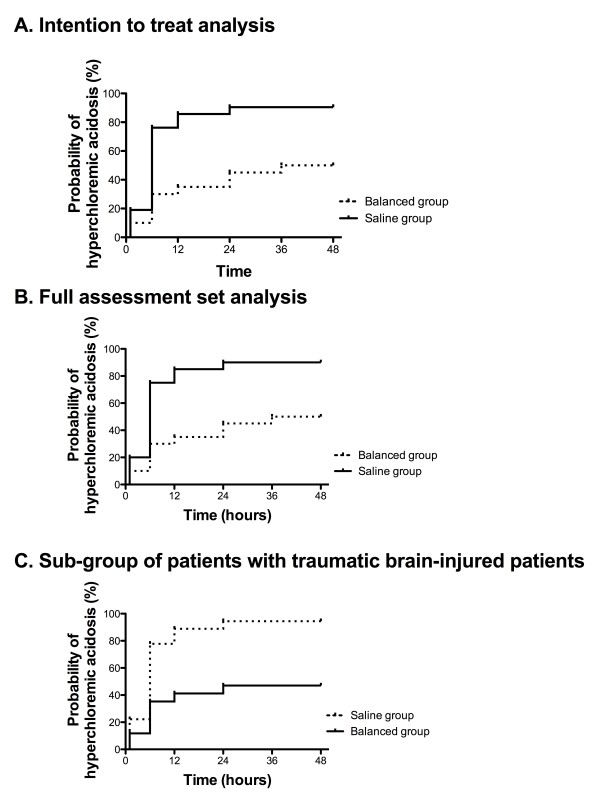
**Kaplan-Meier curves for hyperchloraemic acidosis**. *Hyperchloraemic acidosis *was defined as the association of hyperchloraemia (>108 mmol/L) with strong ion difference (SID) (<40 mmol/L). SID = (Na + K + Ca + Mg) - (Cl + lactate). Na; sodium, K; potassium; Ca: calcium; Mg: magnesium; Cl: chloride.

In the ITT population, 19 patients (90%) in the saline group and 10 patients (50%) in the balanced group had hyperchloraemic acidosis within the first 48 hours (*P *= 0.004). The Kaplan-Meier estimators at hour 48 were 90% (range = 83% to 92%) in the saline group and 50% (range = 31% to 72%) in the balanced group, with a hazard ratio (HR) for hyperchloraemic acidosis in the balanced group of 0.24 (95% CI = 0.10 to 0.59; *P *= 0.003) (Figure 2A).

In the FAS analysis, 18 patients (90%) in the saline group and 10 patients (50%) in the balanced group had hyperchloraemic acidosis within the first 48 hours (*P *= 0.01). The Kaplan-Meier estimators at hour 48 were 90% (range = 73% to 98%) in the saline group and 50% (range = 31% to 72%) in the balanced group, with a HR for hyperchloraemic acidosis in the balanced group of 0.28 (95% CI = 0.11 to 0.70; *P *= 0.006) (Figure 2B). Two sensitivity analyses did not change the results. The HR for hyperchloraemic acidosis in the balanced group was 0.18 (95% CI = 0.06 to 0.55; *P *= 0.002) when the patients with acidosis prior to inclusion were excluded, and it was 0.25 (95% CI = 0.09 to 0.69; *P *= 0.008) with a censorship of the biological values (SID, chloraemia) prior to inclusion. In the subgroup of TBI patients, the HR for hyperchloraemic acidosis in the balanced group was 0.30 (95% CI = 0.12 to 0.80; *P *= 0.015) (Figure 2C).

### Secondary efficacy outcomes

**Figure 3:**
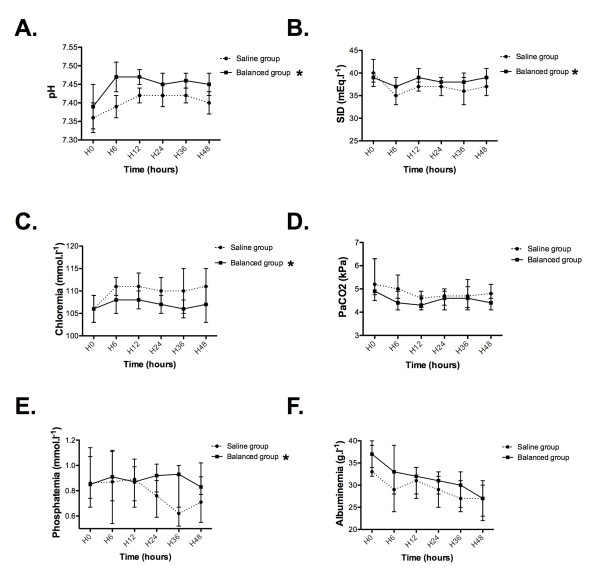
**Time course of acid-base status in the saline group and the balanced group**. *Hyperchloraemic acidosis *was defined as the association of hyperchloraemia (>108 mmol/L) with strong ion difference (SID) <40 mmol/L. SID = (Na + K + Ca + Mg) - (Cl + lactate). **{AU: OK to delete Kaplan? OK Or are words missing?}**According to Stewart *et al*. **(A) **pH is independently influenced by three biological values: first, the SID **(B) **and chloraemia **(C)**; second, the total weak acid concentration composed of phosphor **(D) **and albumin **(E)**; and third, the partial pressure of carbon dioxide in arterial blood (PaCO_2_) **(F)**. Results are given as medians (IQR). *P < 0.05 versus saline group (significant group effect). Na: sodium, K: potassium, Ca: calcium, Mg: magnesium, Cl: chloride.

**Figure 4:**
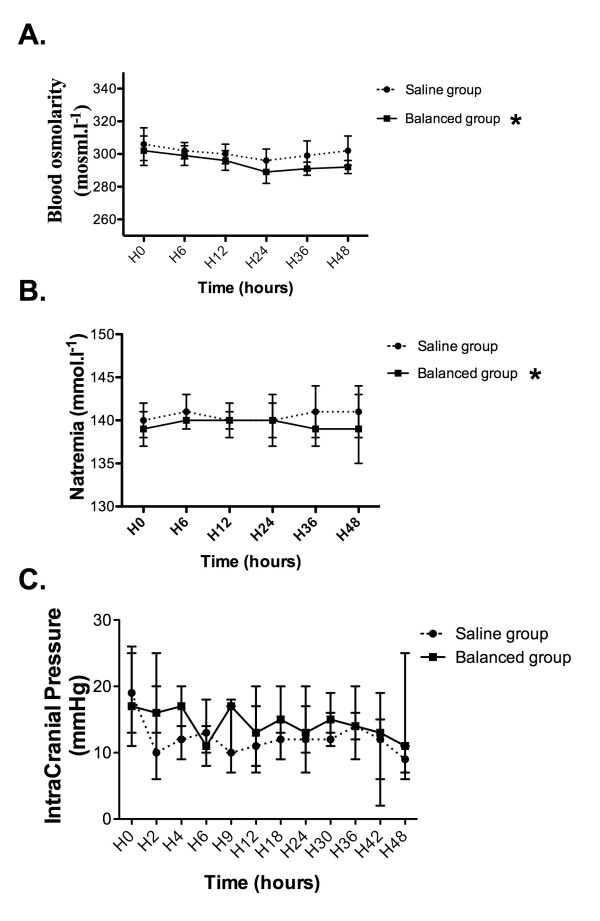
**Time course of (A) blood osmolarity, (B) natraemia and (C) intracranial pressure in the saline group and the balanced group**. Results are given as medians (IQR). *P < 0.05 versus saline group (significant group effect).

The pH was lower in the saline group than in the balanced group (mean difference = -0.03 (-0.05 to -0.01); *P *= 0.004) (Figure 3A). Patients in the saline group had a lower SID than the balanced group (mean difference = -1.55 mEq/L (-3.09 to -0.02); *P *= 0.047) (Figure 3B). Chloraemia was higher in the saline group than in the balanced group (mean difference = 4.8 mmol/L (1.9 to 7.6); *P *= 0.002) (Figure 3C). Compared with the balanced group, patients in the saline group had lower phosphataemia (mean difference = -0.12 mmol/L [-0.21 to -0.04); *P *= 0.008) (Figure 3D). From hour 0 to hour 48, albuminaemia and partial pressure of carbon dioxide in arterial blood (PaCO_2_) were not altered in the saline group compared with the balanced group (Figures 3E and 3F). The reported differences in the acid-base status between groups were not significantly modified in the subgroup of TBI patients (Additional file, Figure S1). The results for base excess, effective SID, simplified anion gap and corrected anion gap are provided in the Additional file, Table S2. The blood osmolarity was higher in the saline group than in the balanced group (mean difference = 7 mOsmol/L (1 to 14); *P *= 0.024) (Figure 4A). The natraemia levels were higher in the saline group than in the balanced group (mean difference = 2 mmol/L (0 to 4); *P *= 0.036) (Figure 4B). ICP was not altered in the study group (mean difference = 4 mmHg (-1 to 8); *P *= 0.088) (Figure 4C). In the subgroup of TBI patients, blood osmolarity, natraemia and ICP were not altered in the study group (see Additional file, Figure S2). Among the entire population, seven patients (35%) in the saline group and eight patients (40%) in the balanced group developed ICH (*P *= 0.744) (Table 4). In the subgroup of patients who developed ICH, ICP was not altered by study group (mean difference = -4 mmHg (-11 to 2); *P *= 0.20). (Additional file, Figure S3).

**Table 4:** Safety assessment^a^

Outcomes	^ITT population^			^TBI patients^		
	
	Saline (*N *= 21)	Balanced (*N *= 20)	*P*-value	Saline (*N *= 18)	Balanced (*N *= 18)	*P*-value
Patients with an episode of ICH, *n *(%)	8 (38)	8 (40)	0.905	6 (33)	8 (44)	0.494
Number of ICH episode per patient, mean ± SD	3 ± 5	1 ± 2	0.912	3 ± 6	1 ± 1	0.957
Management of ICH, *n *(%)						
Osmotherapy (mannitol)	8 (38)	7 (35)	0.837	6 (33)	7 (39)	0.729
Barbiturate	7 (33)	6 (30)	0.819	5 (28)	6 (33)	0.718
Decompressive craniectomy	1 (5)	1 (5)	0.972	1 (6)	1 (6)	1
Therapeutic hypothermia	0 (0)	0 (0)	1	0 (0)	0 (0)	1
Modifications on CT, *n *(%)						
Bleeding	1 (5)	2 (10)	0.52	1 (6)	2 (11)	1
Herniation	0 (0)	1 (5)	0.3	0 (0)	1 (6)	1
Brain swelling	4 (19)	2 (10)	0.413	3 (17)	1 (6)	0.602
In ICU transfusion, *n *(%)						
Red blood cells	7 (33)	4 (20)	0.336	7 (39)	4 (22)	0.278
Fresh frozen plasma	5 (24)	1 (5)	0.089	5 (28)	1 (6)	0.178
Duration of norepinephrine infusion, days, median (IQR)	5 (2 to 7)	4 (2 to 7)	0.676	3 (1 to 4)	4 (2 to 6)	0.503
Potassium administration, *n *(%)	13 (60)	11 (55)	0.654	10 (56)	10 (56)	1
Calcium administration, *n *(%)	4 (20)	2 (10)	0.661	3 (17)	2 (11)	1
Azotaemia, (mol/L), median (IQR)						
Day 1	59 (56 to 68)	60 (50 to 67)	0.879	64 (57 to 74)	67 (59 to 71)	0.975
Day 2	60 (54 to 65)	56 (46 to 64)	0.289	58 (56 to 67)	62 (52 to 68)	0.734
Diuresis, L/day, median (IQR)						
Day 1	1.7 (1.2 to 2.5)	1.6 (1.1 to 2.1)	0.551	1.6 (1.2 to 2.3)	1.6 (1.2 to 2.0	0.756
Day 2	1.6 (1.0 to 2.6)	1.5 (1.0 to 2.0)	0.845	1.6 (1.0 to 2.2)	1.5 (1.0 to 2.0)	0.851
Time to achieve >50% of goal calories of enteral nutrition, days, median (IQR)	4 (3 to 5)	3 (3 to 11)	0.911	4 (3 to 6)	3 (3 to 11)	1
Duration of mechanical ventilation, days, median (IQR)	12 (5 to 18)	12 (8 to 19)	0.823	10 (2 to 18)	10 (7 to 19)	0.76
ICU length of stay, days, median (IQR)	19 (10 to 24)	16 (8 to 21)	0.521	18 (10 to 24)	14 (7 to 21)	0.591
Death in ICU, *n *(%)	3 (14)	5 (25)	0.387	2 (11)	5 (28)	0.402
Refractory ICH	2 (10)	2 (10)		1 (6)	2 (11)	
Care withdrawal	1 (5)	3 (15)		1 (6)	3 (17)	

^a^CT: computed tomography, ICH: intracranial hypertension, IQR: interquartile range, ITT: intention to treat; TBI: traumatic brain injury.

### Safety assessment

Safety outcomes were assessed for the ITT patient population (Table 4). Decompressive craniectomy was performed before ICU admission in one patient (5%) in the balanced group compared with one patient (5%) in the saline group (*P *= 0.972) (Table 4). Three patients (14%) in the saline group died (two with ICH (10%) and one due to care withdrawal (5%)), compared with five patients (25%) in the balanced group (two patients with ICH (10%) and three patients due to care withdrawal (15%)) (*P *= 0.387). No patients died during the study periodThere is no need to provide this information since it is stated that 3 patients died in the saline group and five in the other group.

## Discussion

The present study shows that balanced solutions, in comparison with isotonic saline solutions, reduced the occurrence of hyperchloraemic acidosis in brain-injured patients. Balanced solutions were not associated with ICP alteration or ICH episodes.

According to Stewart *et al*., hydrogen ion concentration (pH) is independently influenced by three biological variables: (1) PaCO_2_, (2) total weak acid concentration (labelled A_tot_) composed of phosphate and albumin and (3) SID corresponding to the difference between strong cations and strong anions [22]. According to Stewart *et al*.'s concept, sodium chloride solutions are responsible for metabolic acidosis through a decrease in SID [23-25]. Thus, administration of drugs with sodium chloride excipient (such as cloxacillin, midazolam or fentanyl) participate in the decrease in SID, which could partially explain the 50% rate of hyperchloraemic acidosis in the balanced group. The concentration of chloride in 0.9% saline solutions surpasses the normal ranges for blood chloraemia [26]. A correlation between hyperchloraemia and base excess has been described in patients undergoing major surgery [16]. Balanced solutions reduce the risk of hyperchloraemic acidosis in elderly patients undergoing major surgery [27,28]. Our results reveal that balanced solutions decrease the risk of hyperchloraemic acidosis in patients with severe brain injury.

At this time, there is increasing evidence that chloride-rich solutions alter the outcomes of critical ill patients [9]. In animal models, chloride-rich solutions decreased the glomerular filtration rate by inducing renal vasoconstriction [29,30]. In a before-after study, the restriction of chloride-rich solutions was associated with a decrease in kidney failure in critically ill patients [10]. Saline-rich solutions alter the coagulation cascade and increase intraoperative blood loss when compared with balanced solutions [31-34]. Hyperchloraemic acidosis also decreases gastric and pyloric motility and could reduce gastric mucosal perfusion [28]. In the present study, balanced solutions prevented hyperchloraemic acidosis but altered neither kidney function (assessed by diuresis and azotaemia) nor gut motility (assessed by the time to achieve more than 50% of enteral nutrition goal calories).

Prevention of hypo-osmolarity is a major goal for the prevention and treatment of ICH. Indeed, hypo-osmolarity induces brain ischaemia resulting from the swelling of perivascular astrocytic cells and also increases ICP and the volume of brain injury [35]. Thus, administration of hypo-osmolar solutions should be avoided in brain-injured patients [6,7]. The isotonicity of the balanced solutions may authorize their utilization in the neuro-ICU, but few data are available in this setting to date. In the present study, the balanced solutions failed to induce hyperosmolarity, and the blood osmolarity was lower in the balanced group than in the saline group. This lower osmolarity in the balanced group may prove important because the maintenance of normal osmolarity is an asset when caring for the brain-injured patients. However, neither ICP evolution nor the rate of ICH were different between the study groups. These results could be explained by the impact of balanced solutions on chloraemia, which is a key regulator of cell volume [10]. Chloride ion efflux prevents cell swelling in hypotonic media [12,36]. The lower chloraemia observed in the balanced group could have increased the phenomenon of chloride ion efflux, limiting brain swelling despite decreased osmolarity compared with the saline group. According to this hypothesis, it has previously been described that a sodium lactate-based hyperosmolar solution more significantly decreased ICP than an equivalent osmotic load of chloride-rich solution [35]. Prevention of hyperchloraemia appears to be an asset for the prevention of ICH in patients with severe brain injury.

There is a controversy about the safety of HES, particularly regarding its effects on coagulation [37]. These concerns could prove to be important in the setting of brain-injured patients. Recently, the Neuro-Intensive Care and Emergency Medicine (NICEM) Section of the European Society of Intensive Care Medicine consensus document stated that HES is not recommended in the context of brain injury [38]. However, this consensus statement was not available when our study started. Moreover, the doses of HES used in each group in our study were below the maximum daily threshold of 20 ml/kg/day.

This study has several limitations. First, given the small number of included SAH patients, the conclusions are valid mainly for the TBI patients. Second, we did not report any differences between groups regarding side effects of hyperchloraemic acidosis. Third, the reported biological differences may not be clinically relevant. Prolonged infusion of 0.9% saline solution may alter clinical outcomes. Fourth, the balanced solution did not alter neurological recovery, and we cannot rule out the theoretical issue regarding the risk of ICH with balanced solutions. However, this pilot study was not powered to evaluate these endpoints.

## Conclusions

The use of balanced solutions reduces the incidence of hyperchloraemic acidosis in brain-injured patients. ICP evolution and the rate of ICH in brain-injured patients did not appear to be different between groups. The safety and impact of balanced solutions on neurological recovery, as well as the potential side effects of balanced solutions, should be investigated in a large, randomised trial comparing balanced solutions and isotonic saline solutions in TBI patients.

## Key messages

• Balanced solutions decrease the incidence of hyperchloraemic acidosis in patients with severe brain injury compare with saline solutions.

• Balanced solutions decrease natraemia and blood osmolarity in patients with severe brain injury.

• Larger studies are required to investigate the effects of balanced solutions on brain swelling and neurological recovery.

## Abbreviations

CT: computed tomography; GCS: Glasgow Coma Scale; HES: hydroxyethyl starch; HSS: hypertonic saline solution; ICH: intracranial hypertension; ICP: intracranial pressure; SAH: subarachnoid haemorrhage; SID: strong ion difference; TBI: traumatic brain injury; WFNS: World Federation of Neurological Societies.

## Competing interests

Karim Asehnoune and Yvonnick Blanloeil have received honoraria from B Braun Medical for public speaking. The other authors have no conflicts of interest to disclose.

## Authors' contributions

All of the authors participated in the study management, data collection and interpretation of data. OL, AR, CL, YB and KA were responsible for the conception and design of the study, interpretation of data and/or writing of the report. RC, ER, PJM, RD, AMC and CP were responsible for data collection, data interpretation and/or writing the report. CV performed statistical analysis. LF managed the blinding and the safety of the study solutions. All authors had full access to all of the data in the study and participated in the revision of the manuscript. All authors read and approved the manuscript for publication.

## Supplementary Material

Additional file 1 Enteral Nutrition Protocol
Table S1. Baseline characteristics
**Table S2. Time evolution of biological values within the first 48 hours **Simplified anion gap (sAG) = Na - (Cl + HCO_3_). Corrected anion gap (cAG) = sAG + 0.25 × (40 - albumin). Effective strong ion difference effective (SIDe) = HCO_3 _+ albumin × (0.123 × pH -0.631) + phosphor × (0.309 × pH -0.469). Data are expressed as median (IQR). **##**Data with a significant interaction between time effect and group effect, comparisons were performed independently for each time point, and *P *values were provided at each time point.
**Figure S1 Time course of acid-base status in TBI patients**. Results are given as median (IQR). *P < 0.05 versus saline group (significant group effect). TBI: traumatic brain injury.
**Figure S2 Time course of (A) blood osmolarity, (B) natraemia and (C) intracranial pressure in traumatic brain-injured patients**. Results are given as medians (IQR).
**Figure S3 Time course of intracranial pressure in brain-injured patients who developed intracranial hypertension**. Results are given as medians (IQR). Click here for file
