# Supplementary material for: Balanced versus chloride-rich solutions for fluid resuscitation in brain-injured patients: a randomised double-blind pilot study
Source: Crit Care. 2013 Apr 19;17(2):R77. doi: 10.1186/cc12686 (PMC4057192; doi:10.1186/cc12686)
Supplement: Additional file 1 — Enteral Nutrition Protocol Table S1. Baseline characteristics Table S2. Time evolution of biological values within the first 48 hours Simplified anion gap (sAG) = Na - (Cl + HCO3). Corrected anion gap (cAG) = sAG + 0.25 × (40 - albumin). Effective strong ion difference effective (SIDe) = HCO3 + albumin × (0.123 × pH -0.631) + phosphor × (0.309 × pH -0.469). Data are expressed as median (IQR). ##Data with a significant interaction between time effect and group effect, comparisons were performed independently for each time point, and P values were provided at each time point. Figure S1 Time course of acid-base status in TBI patients. Results are given as median (IQR). *P < 0.05 versus saline group (significant group effect). TBI: traumatic brain injury. Figure S2 Time course of (A) blood osmolarity, (B) natraemia and (C) intracranial pressure in traumatic brain-injured patients. Results are given as medians (IQR). Figure S3 Time course of intracranial pressure in brain-injured patients who developed intracranial hypertension. Results are given as medians (IQR). [file cc12686-S1.DOCX]

**Supplemental data**

**Balanced versussaline solutions for fluid resuscitation in brain-injured patients.**

**A randomized double-blind controlled trial**

**Enteral Nutrition Protocol**

Enteral nutrition was given according to a written protocol routinely used in our unit. Enteral nutrition was initiated 24 hours after brain injury. Patients were fed continuously. Enteral nutrition preparation was administered through a silicone 14-Fr nasogastric tube inserted by the nurse in charge of the patient. Before starting enteral nutrition, a chest radiograph was used to confirm that the tip of the tube was correctly placed in the stomach. A peristaltic enteral feeding pump injected the enteral nutrition preparation into the tube at a continuous rate. The rate of enteral nutrition delivery was initiated at 20 ml.hour^-1^ and was increased every 8 hours by 20 ml.hour^-1^. The goal was to reach 83 ml.hour^-1^ (2000 Kcal.day^-1^). Tolerance of enteral nutrition was assessed based on repeated residual gastric volume measurements and on records of vomiting episodes. Residual gastric volume was measured at 4-hr intervals by aspirating the nasogastric tube with a 50-mL syringe. The aspirate was returned to the patient unless it exceeded 200 mL. Enteral nutrition was discontinued if the residual gastric volume exceeded 250 mL or if the patient vomited. After discontinuation, prokinetic treatment was instituted (metoclopramide), and enteral nutrition was reintroduced 4 hrs later at the slower rate well tolerated before discontinuation.

**Table S1: Baseline characteristics**

| **Characteristics** | **FAS population** | |
| --- | --- | --- |
|  | **Saline (N=20)** | **Balanced (N=20)** |
| Age, *years, median (IQR)* | 53 (28 – 68) | 49 (26 – 75) |
| Male, *N (%)* | 14 (70) | 17 (85) |
| Diagnosis, *N (%)*  Traumatic brain injury  Subarachnoid Haemorrhage | 18 (90)  2 (10) | 18 (90)  2 (10) |
| Coma Glasgow Scale on scene, *median (IQR)* | 8 (7 - 9) | 7 (6 – 9) |
| Surgical procedures, *N (%)*  Hematoma / contusion evacuation  Extraventricular drainage | 9 (45)  2 (10) | 4 (20)  2 (10) |
| Transfusion, yes, *N (%)*  red blood cells  fresh frozen plasma | 4 (20)  3 (15) | 3 (15)  0 |
| Norepinephrine infusion on admission, *N (%)* | 8 (40) | 7 (35) |
| Fluid Infusion prior to inclusion, *median (IQR)*  Crystalloids (NaCl 0.9%), ml  Colloids, ml | 500 (500 -1000)  0 (0 - 500) | 1000 (500 - 1500)  0 (0 - 500) |
| Time from brain injury to inclusion, *hours, median (IQR)* | 5 (3 - 6) | 5 (4 - 12) |
| Biological status on inclusion  Hyperchloraemic acidosis on inclusion, *N (%)*  Osmolarity (mOsm.l^-1^), *median (IQR)*  Natremia (mmol.l^-1^), *median (IQR)*  Chloraemia (mmol.l^-1^), *median (IQR)*  Kaliemia (mmol.l^-1^), *median (IQR)*  Ionized calciemia (mmol.l^-1^), *median (IQR)*  Magnesemia (mmol.l^-1^), *median (IQR)*  Phosphoremia (mmol.l^-1^), *median (IQR)*  Lactatemia (mmol.l^-1^), *median (IQR*  Azotemia (μmol.l^-1^), *median (IQR)*  Albuminemia (g.l^-1^), *median (IQR)*  pH, *median (IQR)*  SID (mmol.l^-1^), *median (IQR)* | 4 (20)  306 (298 - 319)  140 (138-142)  106 (105 - 110)  3.7 (3.4 - 3.9)  1.10 (1.08 - 1.14)  0.82 (0.72 - 0.92)  0.86 (0.67 - 1.07)  1.5 (1.1 - 2.1)  64 (55-73)  33 (32 - 40)  7.36 (7.33 - 7.42)  40 (36 - 42) | 2 (10)  302 (296 - 319)  139 (137-141)  106 (101 - 107)  3.7 (3.5 - 4.1)  1.11 (1.07 - 1.18)  0.81 (0.75 - 0.88)  0.85 (0.74 - 1.14)  1.7 (1.1 - 2.7)  67 (58-71)  37 (34 - 39)  7.39 (7.32 - 7.45)  39 (39 - 41) |
|  |  |  |

Strong Ion Difference (SID) = (Na^+^ + K^+^ + Ca^2+^ + Mg^+^) - (Cl^-^ + lactates), TBI: Traumatic brain-injured

FAS: full assessment set analysis

**Table S2: Time evolution of biological values within the first 48 hours.**

|  | **Time point** | **Entire population** | | | **TBI patients** | | |
| --- | --- | --- | --- | --- | --- | --- | --- |
|  |  | **Saline group**  **N=20** | **Balanced group**  **N=20** | ***P*-value** | **Saline group**  **N=18** | **Balanced group**  **N=18** | ***P*-value** |
| Excess base (mmol/l) **##** | H0 | -1.7 (-3.9 - -0.3) | -2.6 (-3.5 - -0.1) | 0.004  0.006  0.049  0.263  0.145 | -1.5 (-3.7 - -0.1)  -2.4 (-3.5 - -0.4)  -2.2 (-2.9 - -1.3)  -1.5 (-3.2 - -0.2)  -0.7 (-3.2 - 0.1)  -0.8 (-3.0 - 0.6) | -2.8 (-3.9 - 0.0)  -0.2 (-1.1- 1.0)  0.55 (-0.9 - 1.5)  0.2 (-1.6 - 1.2)  0.1 (-0.7 - 1.0)  -0.6 (-1.3 - 0.5) | 0.019 |
|  | H6 | -2.4 (-3.7 - -0.9) | -0.3 (-1.3 - +1.0) |  |  |  |  |
|  | H12 | -2.2 (-2.9 - -1.3) | +0.4 (-0.9 - +1.3) |  |  |  |  |
|  | H24 | -1.5 (-3.4 - +0.0) | +0.1 (-1.7 - +1.1) |  |  |  |  |
|  | H36 | -1.4 (-3.2 - -0.1) | -0.3 (-1.6 - +1.0) |  |  |  |  |
|  | H48 | -1.0 (-3.0 - +0.1) | -0.6 (-1.4 - +0.4) |  |  |  |  |
| sAG  (mEq/l) | H0 | 10 (8 – 12) | 12 (10 – 14) | 0.011 | 10 (8 - 13)  7 (5 - 9)  8 (6 - 10)  8 (7 - 9)  8 (6 - 10)  8 (6 - 9) | 12 (10 - 14)  10 (8 - 12)  9 (8 - 11)  9 (7 - 10)  8 (6 - 10)  7 (6 - 9) | 0.037 |
|  | H6 | 7 (5 – 9) | 11 (8 – 12) |  |  |  |  |
|  | H12 | 8 (6 – 10) | 10 (8 – 12) |  |  |  |  |
|  | H24 | 8 (7 – 9) | 9 (7 – 11) |  |  |  |  |
|  | H36 | 8 (6 – 10) | 9 (8 – 12) |  |  |  |  |
|  | H48 | 8 (6 – 10) | 9 (7 – 10) |  |  |  |  |
| cAG  (mEq/l) | H0 | 11 (10 – 12) | 13 (11 – 14) | 0.021 | 11 (9-12)  10 (8-11)  11 (9-13)  11 (9-12)  11 (9-13)  11 (10-12) | 13 (11-13)  11 (10-13)  11 (10-13)  11 (10-12)  12 (10-13)  12 (11-13) | 0.045 |
|  | H6 | 10 (8 – 11) | 11 (10 – 13) |  |  |  |  |
|  | H12 | 10 (9 – 12) | 11 (10 – 13) |  |  |  |  |
|  | H24 | 11 (10 – 12) | 11 (10 – 12) |  |  |  |  |
|  | H36 | 11 (9 – 13) | 11 (10 – 13) |  |  |  |  |
|  | H48 | 12 (10 – 13) | 12 (10 – 13) |  |  |  |  |
| SIDe  (mEq/l) **##** | H0 | 34 (31 – 36) | 34 (33 – 36) | 0.003  0.001  0.024  0.063  0.229 | 34 (31-36)  32 (31-34)  32 (29-34)  31 (29-33)  32 (29-34)  32 (29-34) | 35 (32-36)  34 (33-35)  34 (32-35)  34 (31-35)  34 (32-35)  31 (30-34) | 0.037 |
|  | H6 | 32 (31 – 34) | 34 (33 – 35) |  |  |  |  |
|  | H12 | 32 (29 – 34) | 34 (32 – 35) |  |  |  |  |
|  | H24 | 31 (29 – 33) | 34 (32 – 35) |  |  |  |  |
|  | H36 | 31 (29 – 34) | 34 (32 -35) |  |  |  |  |
|  | H48 | 32 (29 – 33) | 31 (30 – 34) |  |  |  |  |

Simplified Anion Gap (sAG) = Na - (Cl + HCO3)

Corrected Anion Gap (cAG) = sAG + 0.25 x ( 40 - albumin)

Strong Ion Difference effective (SIDe) = HCO3 + albumin x (0.123 x pH-0.631) + phosphore x (0.309 x pH-0.469)

Data are expressed as median (IQR)

**##** Data with a significant interaction between time effect and group effect, comparisons were performed independently for each time point and *P* values were provided at each time point.

**Figure S1: Time course of acid-base status in traumatic brain-injured patients.**

Results are given as median (IQR).

* *P*<0.05 versussaline group (significant group effect).

**Figure S2: Time course of (A) blood osmolarity, (B) natraemia and (C) intracranial pressure in traumatic brain-injured patients.**

Results are given as median (IQR).

**Figure S3: Time course of intracranial pressure in brain-injured patients who developed intracranial hypertension.**

**
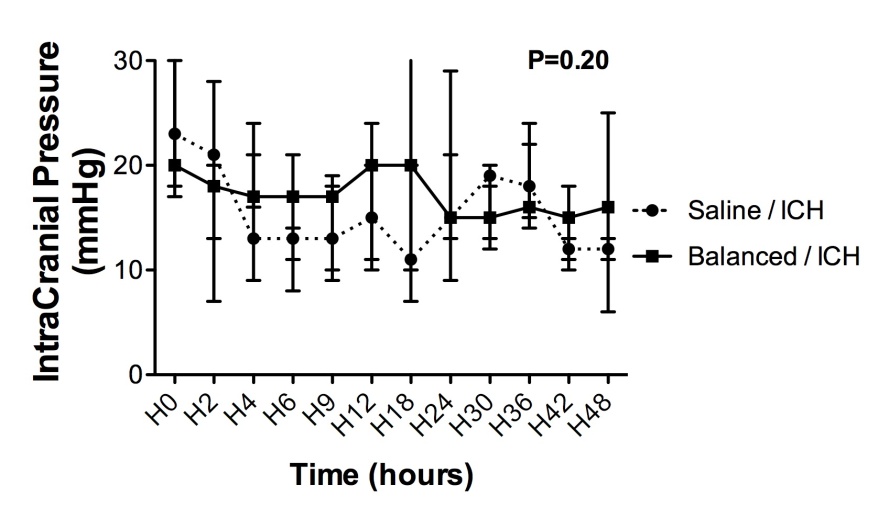
**

Results are given as median (IQR).
